# Er: YAG laser-assisted minimally invasive non-surgical technique (MINST) for periodontal regeneration of maxillary canine teeth in dogs

**DOI:** 10.3389/fvets.2025.1717019

**Published:** 2026-01-14

**Authors:** Kimiyoshi Okano

**Affiliations:** Yokohama Animal Dental Clinic, Kanagawa, Japan

**Keywords:** canine periodontal disease, Er: YAG laser, microscope-assisted dentistry, MINST, periodontal regeneration

## Abstract

Periodontal regeneration around maxillary canine teeth in dogs remains challenging because of limited surgical access and the risk of postoperative complications associated with flap surgery. This article presents a minimally invasive, flapless protocol for periodontal regeneration using an Er: YAG laser-assisted minimally invasive non-surgical technique (MINST). The method enables efficient removal of granulation tissue, root surface conditioning, and blood-clot stabilization under microscopic magnification, without flap elevation. Detailed materials, equipment, and procedural steps are described to support reproducibility. Two representative clinical applications are summarized, showing complete reduction of probing pocket depth and stable healing up to 10 months. This technique may serve as a reproducible, low-invasive alternative to conventional flap-based regenerative surgery for preservation of the maxillary canine teeth in dogs.

## 1 Introduction

Periodontal disease (PD) is one of the most common diseases in companion animals. Epidemiologic studies have shown that the majority of adult dogs are affected by some degree of periodontal disease (1). Progression of PD may result not only in oral complications such as oronasal fistula, jaw fracture, and osteomyelitis, but also in systemic involvement, including associations with cardiac, hepatic, and renal diseases (1). Diagnosis is based on probing and dental radiography, and severity is classified from gingivitis (PD1) to severe periodontitis (PD4). In PD4, preservation of the affected tooth is generally difficult, and extraction is recommended (2).

In veterinary practice, however, preservation of so-called strategic teeth—including canines, maxillary fourth premolars, and mandibular first molars—is recommended prior to considering extraction (2). Maxillary canines are of particular concern because progression of PD often leads to oronasal fistula formation, and regenerative therapy before fistula development has significant clinical value (3). Previous reports have described the application of guided tissue regeneration (GTR) in mandibular canines (4) and the combined use of enamel matrix derivatives (EMD) and bone grafting in maxillary canines (5), with favorable outcomes. Nevertheless, flap-based regenerative surgery inevitably requires gingival incision and periosteal reflection, with associated surgical morbidity and potential postoperative complications (3–5).

In human dentistry, minimally invasive non-surgical techniques (MINST) (6) and modified MINST (M-MINST) (7)—using magnification with a dental microscope or loupes—have recently attracted attention. These techniques achieve debridement of granulation tissue and calculus removal under magnification without incision, and have been shown to reduce probing pocket depth (PPD) and improve clinical attachment level (CAL) (6–8).

In addition, the use of lasers in dentistry has advanced. While diode and CO₂ lasers are commonly applied to soft tissues, they carry risks of carbonization and cracking due to excessive heat, limiting their application to hard tissues. In contrast, the Er: YAG laser, operating at 2940 nm with high water absorption, produces minimal heat and can be safely applied to both soft and hard tissues (9). In human dentistry, Er: YAG lasers have been reported to facilitate removal of granulation tissue, root surface decontamination, and stabilization of the blood clot (10–14), and experimental studies in dogs have also confirmed their utility (9).

The present article provides a detailed methodological description of an Er: YAG laser-assisted minimally invasive non-surgical technique (MINST) specifically adapted for periodontal regeneration of the maxillary canine teeth in dogs.

All materials, equipment, and procedural steps are outlined to support reproducibility, and two representative clinical applications are included to demonstrate the expected outcomes and practical feasibility of this technique.

## 2 Materials and equipment

### 2.1 Animals

**Figure 1:**
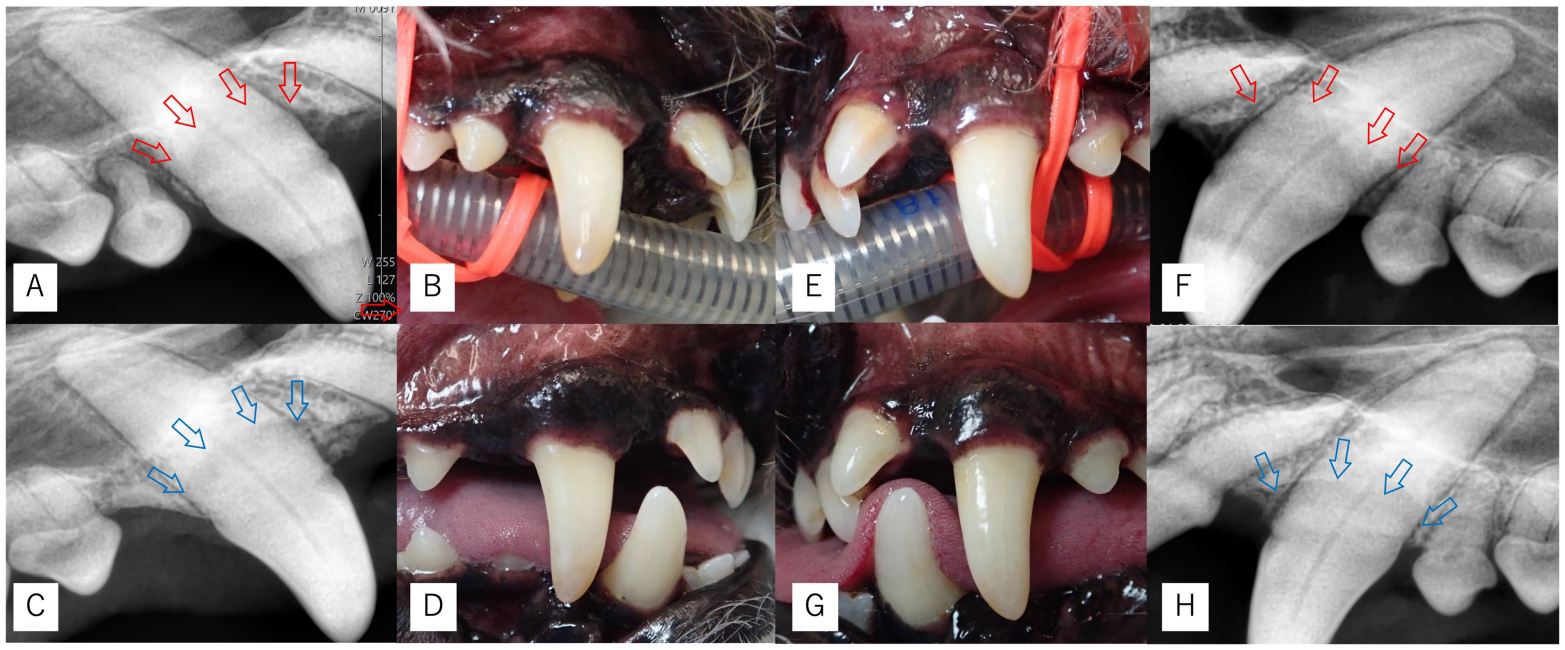
Intraoral and dental radiographic images of Case 1. **(A,F)** Dental radiographs at the initial examination showing palatal bone defects and reduced bone margins (red arrows). **(B,E)** Intraoral photographs at the initial examination. **(C,H)** Dental radiographs at 10 months postoperatively (Day 709) showing maintenance and stabilization of the bone margins (blue arrows), without clearly evident vertical bone height improvement. **(D,G)** Intraoral photographs at the same time point demonstrating good plaque control.

**Figure 2:**
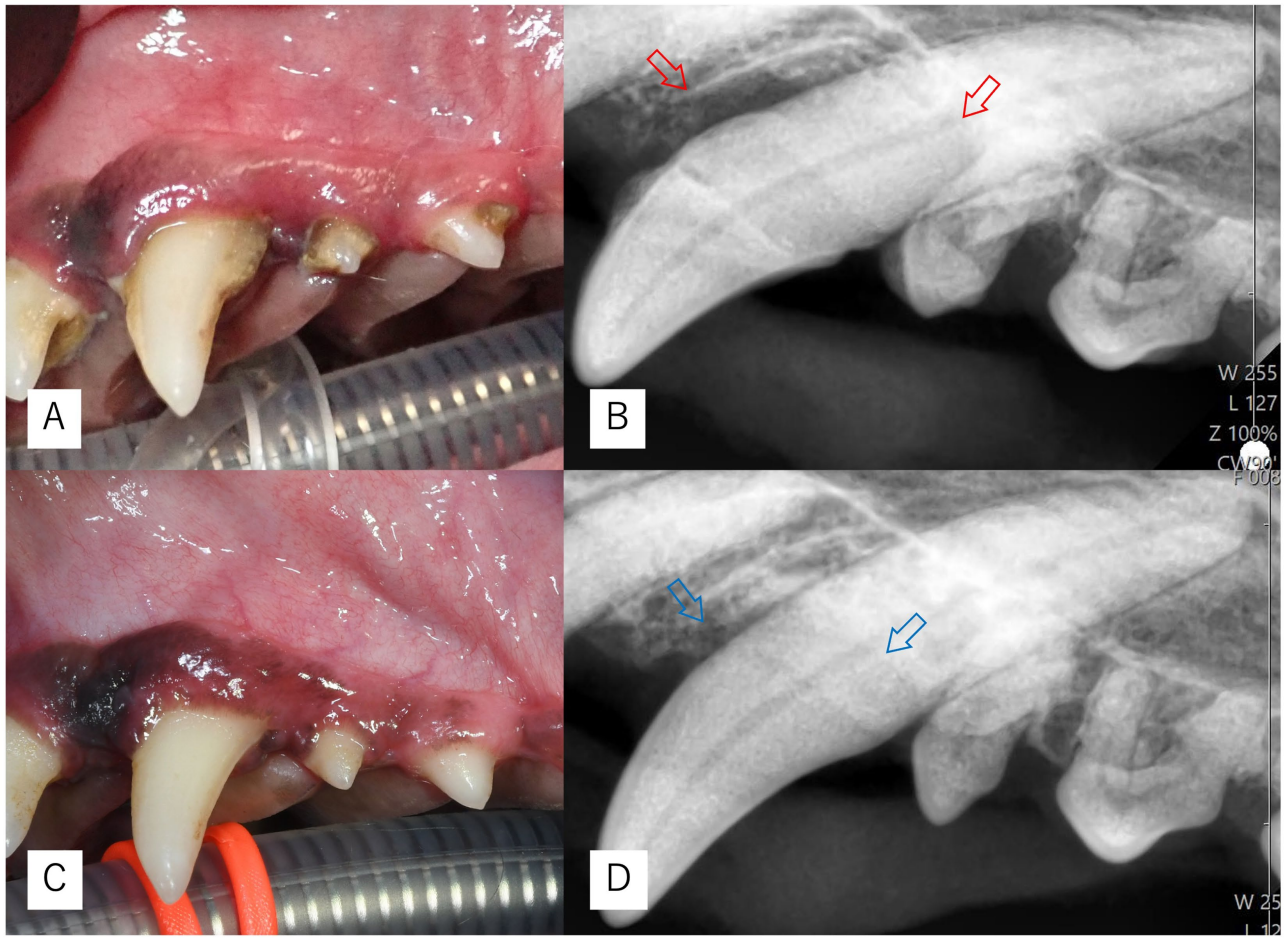
Intraoral and dental radiographic images of Case 2. **(A,B)** Findings at the initial examination showing palatal bone defects (red arrows). **(C,D)** Findings at 6 months postoperatively (Day 195) demonstrating improved bone margins (blue arrows) and favorable plaque control.

Two client-owned dogs—an 8-year-old neutered male Toy Poodle (5.0 kg) and a 7-year-old spayed female Miniature Dachshund (4.5 kg)—presented with advanced periodontal disease affecting the palatal aspects of the maxillary canine teeth. Both animals were systemically healthy based on physical and pre-anesthetic examinations. Informed owner consent was obtained prior to all procedures (Figures 1, 2).

### 2.2 Anesthesia and perioperative management

General anesthesia was induced with intravenous alfaxalone (2 mg/kg; Alfaxan Multidose, Meiji Animal Health, Kumamoto, Japan) following preoxygenation with 100% oxygen. After suppression of laryngeal reflexes, the trachea was intubated using a cuffed spiral-reinforced endotracheal tube (Fuji Systems, Tokyo, Japan), and anesthesia was maintained with isoflurane (ds-Isoflurane, Sumitomo Pharma Animal Health, Osaka, Japan). Cefovecin sodium (8 mg/kg SC; Convenia, Zoetis Japan), meloxicam (0.2 mg/kg SC; Meloxin 0.5%, Fujita Pharmaceutical, Japan), tranexamic acid (10 mg/kg IV; Transamin 10%, Daiichi Sankyo, Japan), and buprenorphine (10 μg/kg IV; Lepetan 0.2%, Otsuka Pharmaceutical, Japan) were administered perioperatively for antimicrobial coverage, anti-inflammatory control, hemostasis, and analgesia.

In addition to general anesthesia, an infraorbital nerve block was performed using 0.25% levobupivacaine (0.15 mL per site; Popsocaine 0.25%, Maruishi Pharmaceutical, Osaka, Japan) to provide regional analgesia and reduce intraoperative nociceptive input.

### 2.3 Instrumentation

**Figure 3:**
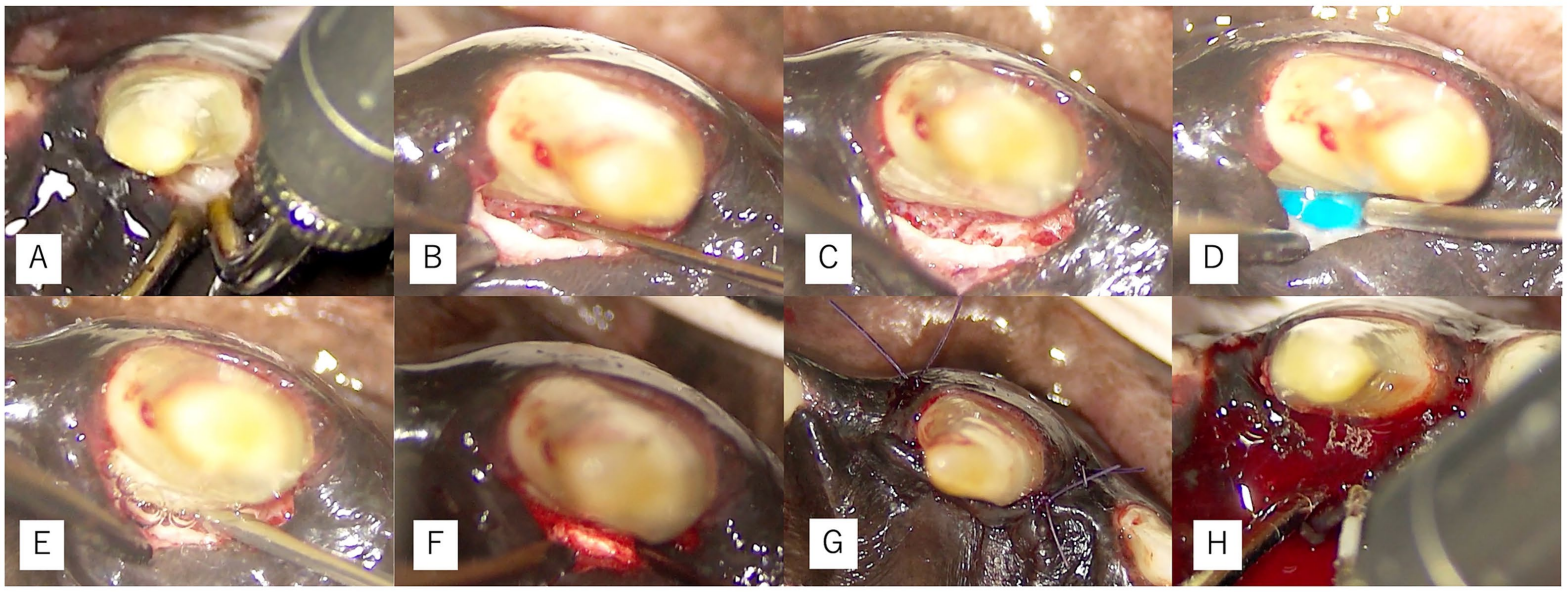
Microscope-assisted surgical procedures in Case 1. **(A)** Er: YAG laser irradiation for debridement of granulation tissue. **(B)** Root debridement using hand curettes. **(C)** Visualization of the osseous defect after debridement. **(D–G)** Adjunctive regenerative procedures for the right maxillary canine, including root surface conditioning with EDTA **(D)**, application of enamel matrix derivative **(E)**, insertion of a collagen plug **(F)**, and placement of simple interrupted sutures **(G)**. **(H)** Laser coagulation for clot stabilization at the left maxillary canine.

All procedures were performed under a dental operating microscope (Bright Vision LED 5103, Pentron Japan, Tokyo, Japan) providing 6–10 × magnification. Scaling and initial plaque removal were performed with an ultrasonic scaler (Cavitron Select SPS, Dentsply Sirona, Tokyo, Japan). The primary instrument for debridement and blood-clot stabilization was an Er: YAG laser (Erwin AdvErL EVO, Morita, Kyoto, Japan). Granulation tissue and residual calculus were removed using both the Er: YAG laser and fine hand curettes (NEW O·K Micro-Exca; Sun Dental, Fukuoka, Japan). For debridement, a PSM600T tip was operated at 20 pulses per second and 50 mJ per pulse with continuous water spray. When clot stabilization was required, defocused irradiation was applied using a C400F tip at 20 PPS and 50 mJ without water spray. Root conditioning was performed with 24% EDTA Blue Gel (EDTA Blue Gel, Yudent, Chiba, Japan), followed by optional application of enamel matrix derivative (Emdogain Gel, Straumann Japan, Tokyo, Japan). A collagen plug (Teruplug, GC, Tokyo, Japan) was inserted as a scaffold when indicated, and wounds were closed with 6–0 monofilament absorbable sutures (Monosyn, B. Braun Aesculap, Tokyo, Japan) where required (Figure 3).

### 2.4 Adjunctive materials and selection criteria

Adjunctive regenerative materials, including enamel matrix derivative (EMD), a collagen plug, and sutures, were not applied uniformly across all treatment sites. Instead, their use was determined intraoperatively based on defect morphology and the stability of the blood clot achieved following laser-assisted debridement.

For sites exhibiting deep vertical intrabony defects (probing pocket depth ≥ 9 mm), adjunctive measures were applied to enhance clot stability and support a favorable regenerative environment. Accordingly, EMD, a collagen plug, and simple interrupted sutures were used in Case 1 (the right maxillary canine teeth) and Case 2 (the left maxillary canine teeth). The collagen plug was inserted from the palatal aspect and gently adapted into the vertical component of the intrabony defect. The plug covered most of the defect; however, due to its size, slight gaps remained at the proximopalatal and distopalatal extensions. The plug was placed to stabilize the blood clot rather than to completely fill the entire defect.

In contrast, Case 1 (the left maxillary canine teeth) presented with a shallower defect (PPD 6 mm), and stable clot formation was achieved through Er: YAG laser coagulation alone. Therefore, no adjunctive materials were applied in this site.

Although these decisions were not based on strict evidence-based thresholds, they reflected consistent clinical judgment regarding defect severity and clot stability and were applied uniformly across cases according to these principles.

### 2.5 Imaging and assessment

Periodontal assessment was performed under anesthesia before and after treatment. Probing pocket depth (PPD) and bleeding on probing (BOP) were measured at six standard site per tooth using a calibrated CPUNC15 probe (CPUNC15, Hu-Friedy Japan, Tokyo, Japan). Dental radiographs were obtained with a portable X-ray generator (EzRay Air, RayVision, Saitama, Japan) and a digital sensor (HDI-1000/2.0, RayVision, Saitama, Japan) to evaluate bone level and defect morphology. All measurements were conducted by the same operator to minimize variability. Owners were instructed to perform daily home plaque control by brushing the teeth once a day with a soft-bristled toothbrush designed for dogs; both dogs were maintained on dry food and did not receive dental chews.

Before treatment, the absence of an oronasal fistula was assessed by periodontal probing for nasal bleeding and by a saline irrigation test confirming no fluid discharge from the nostrils.

During the procedure, granulation tissue was carefully removed under microscopic magnification, allowing direct visualization of the osseous defect and confirmation that no apparent communication with the nasal cavity was present.

Although these evaluations ruled out any clinically evident oronasal fistula, very small or partial-thickness communications cannot be completely excluded.

Although probing pocket depth (PPD) was recorded at six standard sites per tooth, the palatal aspect represented the primary area of clinical interest because the periodontal defects in all treated teeth were located predominantly on the palatal side. For data presentation, the deepest PPD—consistently located on the palatal aspect—was used as the representative value in Tables 1, 2.

**Table 1:** Probing pocket depth (PPD) and bleeding on probing (BOP) of the right and left maxillary canine teeth in Case 1.

Time point	Tooth	Aspect	PPD (mm)	BOP
Baseline (Day 0)	Right maxillary canine	Palatal	7	+
	Left maxillary canine	Palatal	6	+
Re-evaluation (Day 396)	Right maxillary canine	Palatal	9	+
	Left maxillary canine	Palatal	6	+
Post-op 3 months (Day 497)	Right maxillary canine	Palatal	1	−
	Left maxillary canine	Palatal	1	−
Post-op 10 months (Day 709)	Right maxillary canine	Palatal	1	−
	Left maxillary canine	Palatal	1	−

PPD and BOP were recorded at six site per tooth (mesiobuccal, midbuccal, distobuccal, mesiopalatal, midpalatal, and distopalatal); the maximum PPD value for each tooth is presented.

**Table 2:** Probing pocket depth (PPD) and bleeding on probing (BOP) of the left maxillary canine tooth in Case 2.

Time point	Tooth	Aspect	PPD (mm)	BOP
Baseline (Day 0)	Left maxillary canine	Palatal	10	+
Post-op 6 months (Day 195)	Left maxillary canine	Palatal	1	−

PPD and BOP were recorded at six site per tooth; the maximum PPD value for each tooth is presented.

## 3 Results

The following representative applications demonstrate the expected clinical and radiographic outcomes of the Er: YAG laser-assisted MINST protocol.

### 3.1 Representative application – case 1 (toy poodle)

In Case 1, at baseline (Day 0), the palatal probing pocket depths (PPD) of the right and left maxillary canine teeth were 7 mm and 6 mm, respectively, both with bleeding on probing (BOP +) (Table 1), and initial non-surgical periodontal therapy, including scaling and debridement, was performed.

At re-evaluation on Day 396, the right maxillary canine showed progression to a deep vertical intrabony defect with a probing pocket depth of 9 mm.

Based on these findings, Er: YAG laser-assisted MINST was subsequently performed.

The right maxillary canine therefore received adjunctive enamel matrix derivative (EMD), a collagen plug, and simple interrupted sutures.

In contrast, the left maxillary canine had a shallower defect (PPD 6 mm), and stable clot formation was achieved with laser coagulation alone; thus, no EMD, collagen plug, or sutures were applied at this site. Following Er: YAG laser-assisted MINST, both sites showed marked reduction of PPD to 1 mm with BOP (−) at 3 months, which remained stable through 10 months (Table 1). Radiographic and intraoral findings are shown in Figure 1, confirming bone fill and maintenance of gingival contour.

### 3.2 Representative application – case 2 (miniature dachshund)

At baseline, the palatal aspect of the left maxillary canine exhibited a PPD of 10 mm with BOP (+), without mobility. After Er: YAG laser-assisted MINST, PPD was reduced to 1 mm with BOP (−) at 6 months (Table 2). Radiographic and intraoral views in Figure 2 show complete resolution of the intrabony defect and excellent plaque control at follow-up. Because this site presented with a deep vertical defect (PPD 10 mm), adjunctive regenerative measures were used in addition to Er: YAG-assisted MINST. Specifically, enamel matrix derivative (EMD) and a collagen plug were applied, and the site was closed with simple interrupted sutures to enhance clot stability.

## 4 Discussion

The present method demonstrates that flapless periodontal regeneration of the maxillary canine teeth can be achieved through a reproducible combination of minimally invasive non-surgical technique (MINST) and Er: YAG laser therapy. Compared with conventional flap-based regenerative surgery (3–5), this approach minimizes tissue trauma, and reduces postoperative discomfort or risk of wound dehiscence. These advantages are especially relevant for the maxillary canine region, where palatal anatomy restricts surgical access and oronasal fistula formation is a major concern in advanced periodontal disease (1, 2).

In human dentistry, both MINST and its modification (M-MINST) have shown predictable pocket reduction and clinical attachment gain with less morbidity (6–8). Incorporating the Er: YAG laser into this concept provides additional biological benefits: precise removal of granulation tissue, root surface decontamination, bactericidal effects against periodontopathic bacteria, and stabilization of the blood clot (9–14). Experimental studies in dogs have further confirmed that Er: YAG laser irradiation promotes rapid epithelial healing and new bone formation (9). The protocol presented here adapts these advantages into a standardized, replicable workflow optimized for clinical veterinary use.

Microscope-assisted visualization enables controlled debridement of deep palatal pockets without unnecessary removal of healthy tissue. This precise field control, combined with water-cooled Er: YAG ablation, limits thermal damage and preserves the integrity of the gingival margin. Defocused Er: YAG irradiation further stabilizes the blood clot, providing a biological seal comparable to that achieved by suturing, yet without flap tension. These characteristics reduce postoperative inflammation and allow earlier resumption of normal mastication, including prehension and comfortable use of the canine teeth.

Several factors appear critical for successful outcomes. Complete removal of inflamed tissue under magnification and consistent root conditioning establish a clean, biologically receptive surface. Adequate clot stability, achieved by laser coagulation or gentle suturing, protects the regenerative space from bacterial contamination. Equally important is strict postoperative plaque control—daily toothbrushing by the owner—which sustains a low-plaque environment necessary for long-term stability. The two representative cases illustrated that when these principles are respected, even pockets exceeding 6 mm can return to physiologic depth and remain stable for up to 10 months. In addition to these core components, the adjunctive regenerative materials used in some sites—such as enamel matrix derivative, collagen plugs, and simple interrupted sutures—may also have contributed to establishing a stable regenerative environment. Although these materials were applied selectively based on defect severity rather than uniformly across all cases, their potential supportive role should be acknowledged as part of the overall success factors of this protocol.

Despite these advantages, certain limitations must be acknowledged. Although adjunctive regenerative materials were not applied uniformly across all treated teeth, these differences reflected clinical decision-making based on defect morphology and severity rather than methodological inconsistency. Importantly, the core components of the Er: YAG laser–assisted MINST protocol—including microscope-assisted debridement, laser parameters, root conditioning, and postoperative plaque control—were identical in all cases, supporting the methodological integrity of this case series.

The method may be less effective in wide, horizontal defects or in dogs with inadequate owner compliance. Operator experience with magnification and laser handling strongly influences outcome, indicating a learning curve for beginners. Furthermore, this study did not include histologic confirmation of true regeneration, and the small number of clinical applications limits statistical interpretation.

Although the present cases demonstrated marked reductions in probing depth and radiographic evidence of increased bone density, it must be emphasized that the true mode of healing cannot be determined without histologic evaluation. In all treated teeth, the gingival margin remained stable with no postoperative recession or coronal rebound, indicating that the improvements in PPD were not merely the result of soft-tissue repositioning. While it is not possible to distinguish clinically between periodontal regeneration and healing through a long junctional epithelium, the combination of stable gingival architecture and radiographic bone fill suggests that the observed changes reflect genuine periodontal repair rather than an apparent improvement due solely to soft-tissue adaptation.

In addition, this study relied solely on two-dimensional intraoral radiographs, which limit the ability to accurately characterize the precise morphology and location of periodontal defects—particularly on the palatal aspect of the maxillary canine teeth, where access is inherently restricted. Because subtle palatal bone loss or very early oronasal fistula formation may not be fully detectable using 2D imaging, the presence of extremely small or subclinical communications cannot be entirely excluded, even though no clinical signs of oronasal fistula were observed in any case.

Increased radiopacity within the defect area was documented; however, 2D imaging cannot distinguish definitively between new bone formation, defect fill, or changes in mineral density. These radiographic findings should therefore be interpreted as evidence of periodontal repair rather than proof of histologic regeneration. Future research should incorporate three-dimensional imaging such as CBCT, and ideally histologic evaluation, to more precisely characterize the biological nature of the healing response. Controlled clinical trials comparing Er: YAG-assisted MINST with conventional regenerative approaches (3–5), together with detailed radiographic and histological assessments of new cementum and bone formation, will be essential to evaluate the true regenerative potential of this protocol. In addition, comparisons with minimally invasive surgical techniques using enamel matrix derivatives or guided tissue regeneration, as well documented in human periodontology (15, 16), may further clarify the relative advantages of flapless versus surgical regenerative strategies. From a clinical standpoint, this protocol could be integrated into general veterinary dental practice as a conservative option for preserving strategic teeth, reducing the need for extractions, and maintaining oral function and aesthetics. In addition, the flapless design makes the procedure feasible for older or systemically compromised patients, in whom surgical morbidity should be minimized.

In summary, Er: YAG laser-assisted MINST offers a reproducible, low-invasive, and biologically sound protocol for periodontal regeneration of maxillary canine teeth in dogs. It bridges the gap between conventional non-surgical therapy and regenerative surgery, potentially redefining the clinical approach to advanced periodontal disease in veterinary dentistry.

## Data Availability

The original contributions presented in the study are included in the article/supplementary material, further inquiries can be directed to the corresponding author.
